# Disability and COVID-19

**Published:** 2020-09-01

**Authors:** Manfred Mörchen, Harpreet Kapoor, Sara Varughese

**Affiliations:** 1 Ophthalmologist: CBM Regional Advisor for Inclusive Eye Health Asia, Bensheim, Germany.; 2 Director of Ophthalmology & CBM Eye Health Advisor, Max Superspeciality Hospital, Punjab, India.; 3 Country Director & Managing Trustee: CBM, Charmarajpet, Bengaluru, Karnataka, India.

**Several organisations have already published guidelines for eye health services during this pandemic. However, most of them neglect the needs of people with disabilities, including people who are blind or partially sighted.**

**Figure:**
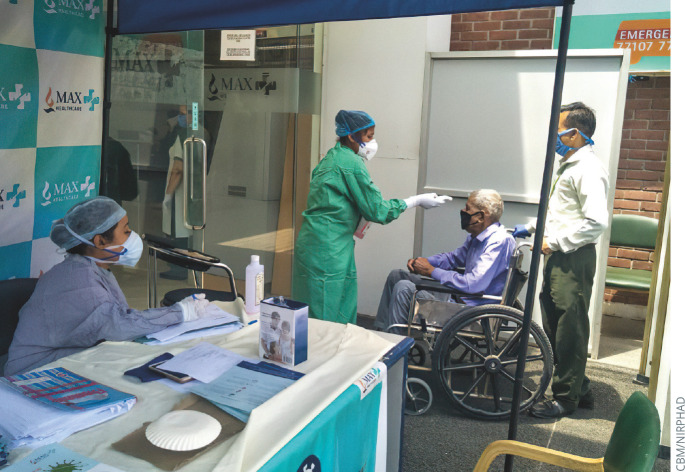
A patient is screened for COVID-19 before being allowed into the eye hospital. **INDIA**

People with disabilities are at greater risk of having difficulties when performing essential hygiene measures (for example, accessing water pumps for handwashing), following recommendations for physical distancing, and/or accessing health information material.^1^ This increases the risk that people with visual impairment and other disabilities may contract SARS-CoV-2.^2^ Here are a few practical recommendations for eye health professionals.

## Involve people with disabilities in decision making

Reach out to local organisations of persons with disabilities (OPD), also known as disabled people’s organisations (DPOs), and involve their representatives in adapting eye health services for people with different types of impairments during the pandemic. If these organisations do not exist, there are very helpful web-based resources available, for example from the International Disability Alliance webpage (International Disability Alliance).^3^

Engaging with Organisations for Persons with Disabilities and including the needs of people with disabilities in the planning and improvement of eye care services should be a long-term goal for eye health services worldwide. For more information, please see the 2013 issue of the *Community Eye Health Journal* titled Disability and Diversity (**www.cehjournal.org/disability-and-diversity/**) and specifically this article: **www.cehjournal.org/article/a-vision-for-inclusion**

## Make communication accessible

Make any mass media communication as accessible as possible by using captioning, sign language translation, high contrast, Braille, and so on.^3^ Short and simple toolkits to improve the accessibility of documents for people with low vision are readily available, for example from the webpage of the World Blind Union.^4^ It is also important to secure a budget for more costly resources, such as sign language translation. The use of face masks, and recommendations to talk as little as possible during slit lamp examinations, significantly reduce verbal and non-verbal communication. It is important to explain the need for these adaptations to patients ahead of an examination.

Face masks might intimidate older patients as well as individuals with hearing, cognitive or psychosocial impairments, and conveying medical information in an encouraging manner is more difficult if patients cannot rely on non-verbal information sources such as facial expression. This can aggravate the general fear and anxiety caused by the pandemic. Be very patient, take additional efforts to repeat information as needed, and try to use transparent masks, if available.

A helpful “ABC mnemonic” was recently published to improve non-verbal communication when wearing facemasks, especially during communication with older patients or those with cognitive impairments: “Attend mindfully – Behave calmly – Communicate clearly.”^5^ Use plain language and clear illustrations to convey health information messages.^6^

## Provide inclusive guidance for people with disabilities

General COVID-19 guidance may not be feasible for people with disabilities. For example, people may not be able to stay 1–2 metres away from others (as recommended by the World Health Organization) if they rely on carers or family members for help with their daily tasks. To “cover your mouth with the elbow when coughing” can be impossible for people with spinal cord injuries or muscular-skeletal conditions. Health information material should inform people about possible modifications and tailored recommendations.^7^ For example, if individuals with cognitive disabilities are not able to avoid touching their eyes, the people supporting them could help them to wash their hands more often. Service providers should make sure that equipment used by people with physical impairments, such as wheelchairs, handrails, and crutches, are frequently cleaned and disinfected, and that people with disabilities have access to water, sanitation and hygiene facilities that do not pose a risk to them.

Some conditions, such as Down’s syndrome, are associated with other health conditions which increase the risk of those affected becoming seriously ill from COVID-19. If possible, proactively test them, and the people supporting and caring for them, for SARS-CoV-2 infection. Carers should also write down who they have been in contact with; this supports contact tracing in communities with lower levels of SARS-CoV-2 infection.

## Hearing impairment

It is very important to consider those patients who are hard of hearing and in need of eye health services, as well as the large number of patients with combined visual and hearing impairments. Results from a population-based study in Telangana state, India, suggested that 25% of people with visual impairment also had an additional moderate or severe hearing impairment.^8^ It is easy to imagine how difficult it might be for them to get information and to communicate with health workers during the COVID pandemic, and all the stress this brings.

Recommendations for medical personnel to facilitate communication with patients who are deaf, hard of hearing or deafblind^9^ include the following:

1. Integrate accessible communication in pandemic preparedness plans. During a pandemic, health systems are overwhelmed. It is essential that medical facilities optimise accessible communication with patients with all types of impairments before a pandemic, so that they are prepared accordingly.
2. Every hospital should have pen and paper, or whiteboards and markers, so that people with hearing impairments can communicate with health care workers.
3. Transparent (see-through) face masks offers speech reading (lip reading) advantages for listeners with severe-to-profound hearing losses, especially in noisy hospital settings.^10^ Instructions for self-made transparent masks are circulating in social media; however, these masks have not been tested against common safety standards, so the level of protection they provide may be no greater than that of a fabric mask.
